# The impact of blood pressure hemodynamics in acute ischemic stroke: a prospective cohort study

**DOI:** 10.1186/1865-1380-5-3

**Published:** 2012-01-17

**Authors:** Latha Ganti Stead, Sailaja Enduri, M Fernanda Bellolio, Anunaya R Jain, Lekshmi Vaidyanathan, Rachel M Gilmore, Rahul Kashyap, Amy L Weaver, Robert D Brown

**Affiliations:** 1 Department of Emergency Medicine, Mayo Medical School, Rochester, MN, USA; 2 Department of Neurology, Mayo Medical School, Rochester, MN, USA; 3 Division of Biostatistics, Mayo Medical School, Rochester, MN, USA; 4 Department of Emergency Medicine, University of Florida, 1329 SW 16th Street, Gainesville, FL, 32610, USA

## Abstract

**Objective:**

To assess relationships between blood pressure hemodynamic measures and outcomes after acute ischemic stroke, including stroke severity, disability and death.

**Methods:**

The study cohort consisted of 189 patients who presented to our emergency department with ischemic stroke of less than 24 hours onset who had hemodynamic parameters recorded and available for review. Blood pressure (BP) was non-invasively measured at 5 minute intervals for the length of the patient's emergency department stay. Systolic BP (sBP) and diastolic BP (dBP) were measured for each patient and a differential (the maximum minus the minimum BP) calculated. Three outcomes were studied: stroke severity, disability at hospital discharge, and death at 90 days. Statistical tests used included Spearman correlations (for stroke severity), Wilcoxon test (for disability) and Cox models (for death).

**Results:**

Larger differentials of either dBP (p = 0.003) or sBP (p < 0.001) were significantly associated with more severe strokes. A greater dBP (p = 0.019) or sBP (p = 0.036) differential was associated with a significantly worse functional outcome at hospital discharge. Those patients with larger differentials of either dBP (p = 0.008) or sBP (0.007) were also significantly more likely to be dead at 90 days, independently of the basal BP.

**Conclusion:**

A large differential in either systolic or diastolic blood pressure within 24 hours of symptom onset in acute ischemic stroke appears to be associated with more severe strokes, worse functional outcome and early death

## Introduction

Stroke is associated with a high mortality and significant long-term functional disability. Of the 15 million patients affected by stroke worldwide yearly, the World Health Organization reports almost a third of these patients die, and another third are permanently disabled. Hypertension accounts for nearly 12.7 million strokes worldwide [1].

Close to 80% of acute ischemic stroke (AIS) patients have an elevated blood pressure [2]. The elevation of blood pressure (BP) post-AIS has a multitude of causes, ranging from chronic hypertension and sympathetic stress response to stroke-related pathology itself [3]. Previous studies have shown that the location of the stroke [4] and the type of stroke [5] have some bearing on the blood pressure response noted acutely post-AIS. Some cohort studies have also suggested that admission blood pressure prognosticates outcome after acute ischemic stroke [6], whereas others conducted similarly have refuted the above result [7,8].

Treatment strategies for hypertension post-AIS are centered on the aim to salvage the ischemic penumbra [9], but the management of hypertension in patients with acute ischemic stroke has been greatly under debate, with no clear consensus on how much or how soon to lower the pressure [10].

It is well known that normally cerebral auto-regulation maintains perfusion over a wide range of systemic blood pressures. During the acute phase of stroke, cerebral auto-regulation becomes dysfunctional [11], and cerebral perfusion pressure becomes directly dependent on systemic pressure. As a result cerebral blood flow becomes passive, with a linear relationship between systemic BP and cerebral blood flow across a wide range of pressure values. Even a relatively small degree of systemic BP reduction could cause a significant risk of hypoperfusion and ischemia [12]. Moreover, there is also impairment of vasomotor tone after AIS [13]. Hence, it has been deliberated whether systemic pressure variation has any consequence on stroke severity, functional disability or death.

Previously published data demonstrate that acute blood pressure variability within early hours of presentation to the emergency department (ED) is associated with an increased risk of death at 90 days [14]. Both very high and very low initial blood pressures are known to be predictors of worse outcomes in AIS [15,16]. With published guidelines recommending permissive hypertension in the early course of AIS [17], and promising results of current research on hemodynamic augmentation in AIS, a cautious approach to treatment of hypertension in AIS is the call of the hour [18].

We sought to determine if there was any association between fluctuations in systolic (sBP) or diastolic (dBP) blood pressure within 24 h of the onset of AIS and stroke severity, functional disability and mortality.

## Methods

The study cohort for this IRB (institutional review board) approved prospective cohort follow-up study consisted of prospectively enrolled consecutive adult patients presenting to our academic ED with AIS. Patients with AIS onset > 24 h prior to presentation/indeterminable time of onset and patients with non-reviewable consecutive vitals were excluded from the final cohort.

Blood pressure (BP) was non-invasively measured at 5-min intervals for the length of the patient's ED stay with the Philips M3046A Patient Monitoring System (Philips Medical Systems, Andover, MA). The system design uses the oscillometric method, measuring the pulsed amplitude of pressure changes in the cuff as it deflates, to demarcate the systolic and diastolic blood pressures. The 24-h differential pressure, defined as the difference between the maximum and the minimum pressures, was calculated for both the sBP and the dBP.

Besides the routine demographics, data on stroke severity on arrival, disability at hospital discharge and death at 90 days were collected for the study cohort. Stroke severity on arrival was measured by the National Institutes of Health Stroke Scale (NIHSS), and disability at discharge was measured by the modified Rankin score (mRS). Poor functional outcome was defined as a mRS ≥ 3 at discharge. Death at 3 months was ascertained by scripted telephone follow-up, state death certificates and electronic medical records with prior patient authorization.

JMP 8.0 was used for the analysis using Spearman correlations (for stroke severity), Wilcoxon test (for disability) and Cox models (for death).

## Results

Demographics and characteristics of the cohort are summarized in Table 1. With 58.7% males, the cohort had a mean age of 74 years (SD = 15.0). A study of the TOAST classification of the type of strokes revealed an unusually high number of cardio-embolic strokes in our cohort (50.3%).

**Table 1:** Study cohort demographics and characteristics

Demographics and characteristics	*N *= 189
Male gender	111 (58.7%)
Age (years)	
Mean (SD)	74.0 (15.0)
Range	26-98
TOAST	
1. Large vessel	20 (10.6%)
2. Cardioembolic	95 (50.3%)
3. Small vessel	29 (15.3%)
4. Other, no causes or multiple causes	45 (23.8%)
NIHSS	
Mean (SD)	9.9 (8.5)
Median (IQR)	6.0 (3.8-15)
Range	0-37
Rankin score at dismissal	
0-2	58 (31.0%)
3-6	129 (69.0%)

The median number of blood pressure readings was 7, with an inter-quartile range (IQR) of 4 to 10. The median systolic blood pressure on arrival (baseline sBP) was 161 mmHg (IQR 144 to 188 mmHg), and the median diastolic blood pressure (baseline dBP) was 80 mmHg (IQR 70 to 90 mmHg). The median diastolic BP differential was 27 mmHg (IQR 16 to 41 mmHg), and the median systolic BP differential was 33 mmHg (IQR 19 to 53 mmHg).

A statistically significant relation was found between baseline hypertension and death at 90 days, when defining baseline hypertension as baseline sBP = 170 mmHg and/or baseline dBP = 110 mmHg. A total of 41.07% patients had baseline hypertension using the above definition. The relative risk of mortality at 90 days for patients with baseline hypertension was 2.05 with a 95% confidence interval of 1.02-4.10 when compared to patients presenting with lower BP (*p *= 0.038).

We also divided the cohort into those with or without one or more episodes of frank hypotension using the minimum measured dBP cutoff of 70 mmHg. Sixty-five percent of the cohort had dBP < 70 mmHg sometime during the stay in ED. When this group was compared to those with dBP > 70 mmHg, there was however no statistical difference in stroke severity, outcomes of death at 90 days or mRS at discharge.

The median NIHSS score at arrival was 6.0, with an interquartile range of 3.75 to 15.0. Patients with more severe strokes had larger differential dBP (*p *= 0.003) and differential sBP (*p *< 0.001) (Spearman correlation *r *= 0.22 and *r *= 0.26, respectively). There was no association found between baseline hypertension and NIHSS score on arrival (*p *= 0.4734).

A total of 129 patients (68.3%) had a Rankin score of 3 or more at hospital discharge (bad outcome). The median differential dBP for a Rankin score 0-2 was 22.5 mmHg, while for a Rankin score of 3 or more was 29 mmHg. The median differential sBP for a Rankin score 0-2 was 26 mmHg, while for a Rankin score of 3 or more was 36 mmHg. A greater dBP (*p *= 0.019) or sBP (*p *= 0.036) differential was associated with a significantly worse functional outcome at hospital discharge (Table 2). Again, as with NIHSS, there was no relation between baseline hypertension and bad outcome (*p *= 0.486).

**Table 2:** Comparison between Rankin score and BP

BP (mmHg)	Rankin score 0-2 (*n *= 41)	Rankin score 3-6 (*n *= 88)	*p*-value
Baseline sBP (mean ± SD)	170.8 ± 35.3	166.5 ± 32.2	0.490
Baseline dBP (mean ± SD)	80.9 ± 21.0	80.2 ± 22.0	0.855
Change sBP (median, 25%-75% IQR)	26.0, 15.5-48.5	36.0, 22.0-53.5	**0.036**
Change dBP (median, 25%-75% IQR)	22.5, 12.0-37.0	29.0, 18.5-45.0	**0.019**

p-values less than 0.05 considered statistically significant are shown in bold.

A total of 40 deaths (21.2%) occurred within the first 90 days. Those with larger differentials of either dBP (*p *= 0.008) or sBP (0.007) were significantly more likely to be dead at 90 days (Table 3). This association retained statistical significance even after adjusting for stroke severity.

**Table 3:** Comparison between mortality and BP

BP (mmHg)	Alive at 90 days (*n *= 149)	Dead at 90 days (*n *= 40)	*p*-value
Baseline sBP (mean ± SD)	168.8 ± 32.4	163.8 ± 34.7	0.449
Baseline dBP (mean ± SD)	80.7 ± 20.3	80.4 ± 25.2	0.956
Change sBP (median, 25%-75% IQR)	30.0, 17.0v51.5	42.5, 29.0-61.0	**0.033**
Change dBP (median, 25%-75% IQR)	25.0, 14.0v38.0	35.0, 23.3-51.8	**0.006**

p-values less than 0.05 considered statistically significant are shown in bold.

## Discussion

Most hemodynamic variables, including systolic blood pressure, diastolic blood pressure, mean arterial pressure pulse pressure and heart rate, have been associated with poor functional outcome following stroke [19]. Like earlier studies, we too found that baseline hypertension was associated with a higher risk of death at 90 days post-stroke, although it was not associated with stroke severity at presentation. For our cohort this hypertension cutoff was a blood pressure of 170/110 mmHg.

With the ongoing discussion on management of blood pressure in acute stage of ischemic stroke, researchers have tried to establish relationships between outcomes and blood pressure. One such study by Toyoda et al. in 2009 reported that systolic blood pressure values between 12 and 36 h post-admission were predictive of neurological deterioration, but the authors did not find the same for blood pressure values within the initial 6 h [20].

Recently concern has been expressed over the relation between higher pre-treatment systolic blood pressure and poor re-canalization in patients treated with IV tPA [21]. Our own research in 2006 revealed that wide fluctuations in blood pressure in the first 3 h of emergency department stay predicted mortality over 3 months post-stroke [14].

This study builds on our prior work on blood pressure and acute ischemic stroke (14, 15), a follow-up to our earlier study. We questioned whether it was indeed the BP differential that resulted in poor outcomes or rather an episode of hypotension during the early ED course that was the culprit. When we compared the hypotensive and non-hypotensive groups, we found that there was no difference in the outcomes of death or functional Rankin scores, suggesting that BP fluctuation was an independent predictor of poor outcome. The VISTA collaboration presented similar findings, highlighting that fluctuations in systolic blood pressure were associated with worse outcome post-stroke [22].

Our study also found that patients with more severe strokes had greater fluctuations in blood pressure, but not the initial baseline blood pressure. This led to the argument that it was the initial severity of the stroke that translated to the worse outcome. However, when we adjusted for the NIHSS severity of strokes, we found that the sBP differential and dBP differential co-related independently with death at 90 days.

Hypotension relative to the baseline, causing regional hypo-perfusion, is an increasingly understood concept immediately following an ischemic stroke. The results of the present study and ensuing discussion may tempt one to surmise that blood pressure variability is bad and that therefore somehow tightly controlling it within a specified range is the next logical step. Caution must be exercised here. One cannot assume that "correcting" the association will result in improved outcome. It is indeed the next step in clinical investigation, but not quite ready for implementation into clinical practice before the hypothesis is definitively investigated in a controlled trial.

## Conclusion

Fluctuations in blood pressure in the setting of acute ischemic stroke appear to impart a negative impact on stroke severity, functional outcome and death at 90 days. This is a hypothesis-generating study that asks whether pharmacologic control of these blood pressure fluctuations would result in improved clinical outcomes.

## Patient consent

This protocol was approved by the department of Emergency Medicine Research Committee (minute excerpt attached). It was also approved by the Mayo Clinic Institutional Review Board as protocol 1054-04.

## Authors' contributions

LGS conceived the study, collected the data and wrote the paper. SE, MFB, AJ, LV, RG, and RK collected data and reviewed the paper. ALW analyzed the data. RDB supervised the project. All authors read and approved the final manuscript.

## Competing interests statements

The authors declare that they have no competing interests.
